# Supplementary material for: tRNA Derivatives in Multiple Myeloma: Investigation of the Potential Value of a tRNA-Derived Molecular Signature
Source: Biomedicines. 2021 Dec 1;9(12):1811. doi: 10.3390/biomedicines9121811 (PMC8698603; doi:10.3390/biomedicines9121811)
Supplement: Supplementary file 1 [file biomedicines-09-01811-s001.zip › Table S1.pdf]

Table S1. Subgrouping of the 76 MM patients based on the levels of each tRF in CD138+ plasma cells.

tRF status	Frequency
**i-tRF-Pro^{TGG}**	
Positive	55 (72.4%)
Negative	21 (27.6%)
**i-tRF-Glu^{CTC}**	
Positive	51 (67.1%)
Negative	25 (32.9%)
**i-tRF-His^{GTG}**	
Positive	54 (71.1%)
Negative	22 (28.9%)
**i-tRF-Gly^{GCC}**	
Positive	34 (44.7%)
Negative	42 (55.3%)
**i-tRF-Phe^{GAA}**	
Positive	48 (63.2%)
Negative	28 (36.8%)
**3′-tRF-Leu$^{AAG/TAG}$**	
Positive	37 (48.7%)
Negative	39 (51.3%)
