# Supplementary material for: tRNA Derivatives in Multiple Myeloma: Investigation of the Potential Value of a tRNA-Derived Molecular Signature
Source: Biomedicines. 2021 Dec 1;9(12):1811. doi: 10.3390/biomedicines9121811 (PMC8698603; doi:10.3390/biomedicines9121811)
Supplement: Supplementary file 1 [file biomedicines-09-01811-s001.zip › Table S2.pdf]

Table 2. Associations between the status of each tRF and the chromosomal aberration (+1q).

tRF status	Total	Chromosomal aberration (+1q)		*P* value[1]
		Presence	Absence	
**i-tRF-Pro^{TGG}**				
Positive	36	18 (50.0%)	18 (50.0%)	0.38
Negative	18	6 (33.3%)	12 (66.7%)	
**i-tRF-Glu^{CTC}**				
Positive	37	18 (48.6%)	19 (51.4%)	0.37
Negative	16	5 (31.2%)	11 (68.8%)	
**i-tRF-His^{GTG}**				
Positive	38	19 (50.0%)	19 (50.0%)	0.24
Negative	16	5 (31.2%)	11 (68.8%)	
**i-tRF-Gly^{GCC}**				
Positive	22	14 (63.6%)	8 (36.4%)	*0.027*[2]
Negative	32	10 (31.2%)	22 (68.8%)	
**i-tRF-Phe^{GAA}**				
Positive	32	17 (53.1%)	15 (46.9%)	0.17
Negative	22	7 (31.8%)	15 (68.2%)	
**3′-tRF-Leu$^{AAG/TAG}$**				
Positive	23	13 (56.5%)	10 (43.5%)	0.17
Negative	31	11 (35.5%)	20 (64.5%)	

[1] Calculated using Fischer's exact test. [2] Italics indicate a significant *P* value.
